# Effect of low-dose atrial natriuretic peptide in critically ill patients with acute kidney injury: a retrospective, single-center study with propensity-score matching

**DOI:** 10.1186/s12882-020-1701-7

**Published:** 2020-01-30

**Authors:** Keita Saito, Shigehiko Uchino, Tomoko Fujii, Shinjiro Saito, Masanori Takinami, Shoichi Uezono

**Affiliations:** 1 Intensive Care Unit, Department of Anesthesiology, The Jikei University School of Medicine, 3-19-18, Nishi-Shinbashi Minato-ku, Tokyo, 105-8471 Japan; 2 The Australian and New Zealand Intensive Care Research Centre, Monash University, 553 St Kilda Rd, Melbourne, VIC 3004 Australia; 3 Graduate School of Medicine, Kyoto University, Yoshida-Honmachi, Sakyo-ku, Kyoto, 606-8501 Japan; 4 Department of Anesthesiology, The Jikei University School of Medicine, 3-19-18, Nishi-Shinbashi Minato-ku, Tokyo, 105-8471 Japan

**Keywords:** Atrial natriuretic peptide, Critically ill patients, AKI treatment

## Abstract

**Background:**

Acute kidney injury (AKI) is a major comorbidity in critically ill patients. Low-dose atrial natriuretic peptide (ANP) has been shown to effectively prevent acute kidney injury (AKI), especially in cardiovascular surgery patients. However, its treatment effects for AKI in critically ill patients are unclear.

**Methods:**

This single-center, retrospective, observational study included patients with AKI diagnosed within 7 days after intensive care unit (ICU) admission during the period January 2010 to December 2017. We conducted a propensity-matched analysis to estimate the treatment effect of low-dose carperitide (a recombinant human ANP) on the clinical outcomes. The primary outcome was a composite of death, renal replacement therapy dependence, or no recovery from AKI (defined as an increase of the serum creatinine level to ≥200% of baseline) at hospital discharge.

**Results:**

During the study period, 4479 adult patients were admitted to the ICU. We identified 1374 eligible patients with AKI diagnosed within 7 days after ICU admission. Among these patients, 346 (25.2%) were treated with low-dose carperitide, with an average dose of 0.019 μg kg^− 1^ min^− 1^. The primary outcome occurred more often in the treatment group than in the control group (29.7% versus 23.4%, respectively; *p* = 0.022). After propensity score matching, characteristics of 314 patients from each group were well- balanced. Significant difference of the primary outcome, as seen with the full cohort, was no longer obtained; no benefit of carperitide was detected in the matched cohort (29.0% versus 25.2%; *p* = 0.281).

**Conclusions:**

Low-dose ANP showed no treatment effect in general critically ill patients who developed AKI.

## Background

Acute kidney injury (AKI) is one of the most common forms of organ damage encountered in the intensive care unit (ICU) and is associated with a high mortality rate [1–3]. Even after discharge from the ICU, the renal function of these patients is often not recovered to the premorbid level [4]. Progression to end-stage kidney disease of AKI patients was reported 3.1 times compared with non-AKI patients [5], which can affect quality of life, both physically and mentally [6]. Although many treatment strategies have been explored to date, none have proved to be effective in improving patient outcome [7].

Atrial natriuretic peptide (ANP) is an endogenous hormone that is released from the atrium. It plays an important role in fluid volume and blood pressure regulation, which has been studied for more than 30 years as a promising drug for AKI [8]. ANP affects the afferent arterioles of the glomerulus more strongly than the efferent arterioles, resulting in increased glomerular filtration rate (GFR) [9]. In addition, ANP exerts anti-inflammatory effects by inhibiting nuclear factor-κB activation and cytokine production [10]. Studies have also reported that ANP can prevent lipopolysaccharide-induced oliguria by activating guanylyl cyclase A in proximal tubules and endothelial cells [11]. These properties make ANP attractive as a potential drug to prevent or treat AKI. Indeed, in patients with ischemic acute renal failure, ANP at an infusion rate of 0.05 μg kg^− 1^ min^− 1^ induced an increase in renal blood flow and GFR by approximately 40% [12, 13]. Several systematic reviews and meta-analyses of ANP have been performed, which showed some beneficial effects of ANP including decreased serum creatinine levels and renal replacement therapy (RRT) requirement and decreased ICU and hospital length of stay [14–16]. However, most of the prior comparative studies looked at the preventive effects of ANP in the context of cardiovascular surgery [17–27] or contrast-induced nephropathy [28–30]. Limited information is available regarding the therapeutic effects of ANP in critically ill patients.

One meta-analysis found that ANP was associated with a trend toward increased mortality and more adverse events when administered in high doses, i.e. > 0.05 μg/kg/min, possibly due to its induction of hypotension [14]. Another meta-analysis focused on low-dose ANP, i.e. < 0.05 μg/kg/min, found a significant decrease in RRT requirement with respect to both prevention and treatment of AKI in post-cardiac surgery patients [16]. However, the beneficial effects of low-dose ANP in patients with AKI in the ICU have not been examined sufficiently [31, 32].

Here, we have assessed the therapeutic effect of low-dose ANP on outcomes of critically ill patients with AKI using a large database.

## Methods

We conducted a single-center, retrospective, observational study in a 20-bed mixed ICU of an academic hospital in Tokyo, Japan. This study was conducted in accordance with the principles of the Declaration of Helsinki, and the ethical committee and institutional review board of the Jikei University Hospital approved the study protocol, No. (30–275 [9296]). Because of its retrospective, observational nature, the committee waived the need for written informed consent.

### Study setting and participants

Patients who were admitted to the ICU from January 1, 2010, through December 31, 2017 were included screened. We identified patients ≥18 years of age with ≥24 h of ICU stay who received a diagnosis of AKI within 7 days after ICU admission. Diagnosis of AKI was made according to the Acute Kidney Injury Work Group Kidney Disease: Improving Global Outcomes (KDIGO) definition [33]. Baseline creatinine level was defined as the mean outpatient serum creatinine value measured 7 to 365 days before hospital admission [34]. If baseline creatinine data were not available, we estimated the level according to the equation for Modification of Diet in Renal Disease (MDRD) for Japanese [35]. We excluded patients with end-stage kidney disease (ESKD), those who had kidney transplantation, and those with a history of urinary diversion. We also excluded patients who did not have AKI during the first 7 days in the ICU or those who received ANP before the diagnosis of AKI. For patients with multiple admissions to the ICU during a single hospitalization period, only the first ICU admission was included.

In Japan, carperitide (HANP®, Daiichi-Sankyo Pharmaceutical Inc., Tokyo, Japan), a recombinant human ANP, is the only ANP agent available commercially. We categorized eligible patients into those received carperitide within 7 days after AKI diagnosis (treatment group) and those did not receive it (control group). The timing and dosage of carperitide administration was determined at the discretion of the treating physician.

### Variables and outcomes

Medical records were reviewed, and following data were collected: age, sex, height, body weight, ICU admission route (operating room [elective or emergency], emergency department, ward, other hospital), comorbidities (hematologic disease, metastatic cancer, immunosuppression, liver failure), primary damaged organ system (cardiovascular, respiratory, digestive, neurologic, other), presence of infection at ICU admission, use of noninvasive positive-pressure ventilation, hours of mechanical ventilation, Acute Physiology and Chronic Health Evaluation (APACHE) II score [36], serum creatinine level (baseline, at ICU admission, and at AKI diagnosis), days from ICU admission to AKI diagnosis and days from AKI diagnosis to ANP administration.

The primary outcome was a composite of hospital mortality, the need of RRT or no recovery from AKI (defined as an increase of the serum creatinine level to ≥200% of baseline) at hospital discharge [37]. Secondary outcomes included the highest AKI stage during ICU stay (AKI max, AKI max-creatinine, AKI max-urine output), RRT use during ICU stay, ICU length of stay (LOS), hospital LOS, ICU mortality, and dialysis-free survival at hospital discharge. We also collected creatinine data at Day 1, Day 2, and Day 3 after AKI diagnosis and at ICU discharge; urine output at 24 h, 48 h, and 72 h after AKI diagnosis; the median dose of carperitide administered and carperitide infusion period.

### Statistical methods

Patient characteristics and outcomes were analyzed for differences between the two groups by the Mann-Whitney *U* test for continuous variables and the Fisher’s exact test or chi square test for categorical variables. We created box plots for creatinine data (at Day 1, Day 2, and Day 3 after AKI diagnosis and at ICU discharge) and urine output (at 24 h, 48 h, and 72 h after AKI diagnosis) divided by the two groups and compared them with the Mann-Whitney *U* test.

We constructed a logistic model for carperitide administration to calculate the propensity score (PS) for each patient on the basis of the following variables: age, sex, body weight, height, source of admission to ICU, primary damaged organ system, APACHE II chronic health condition, APACHE II score, baseline serum creatinine level, days from ICU admission to AKI diagnosis, and serum creatinine level at AKI diagnosis. Propensity score matching using nearest-neighbor method was performed in a 1-to-1 fashion between the treatment group and the control group using calipers of width equal to 0.2 of the standard deviation of the logit of the PS [38]. Covariate balances before and after matching were checked by comparing standardized differences [39]. A standardized difference of < 0.10 was considered to indicate successful balancing. Propensity score matching, calculation of standardized difference and survival analysis were performed using R (version 3.4.3; R Foundation for Statistical Computing, Vienna, Austria). We used ‘Matching’ package for the propensity score matched analysis. For the survival analysis, we used ‘survival’ package and ‘survminer’ package. All other statistical analyses were performed using SPSS (version 19.0; IBM Corp., Armonk, NY, USA). A two-sided *p* value less than 0.05 was considered to have statistical significance.

#### Additional analysis

As post-hoc analysis, Kaplan-Meier survival curves of 90-day death were plotted and compared between groups using the log-rank test. We additionally performed an analysis fitting marginal structural model using inverse probability of treatment weights (IPTW) where standardised weights were estimated. We used ‘ipw’ package in R for the analysis.

## Results

**Fig. 1:**
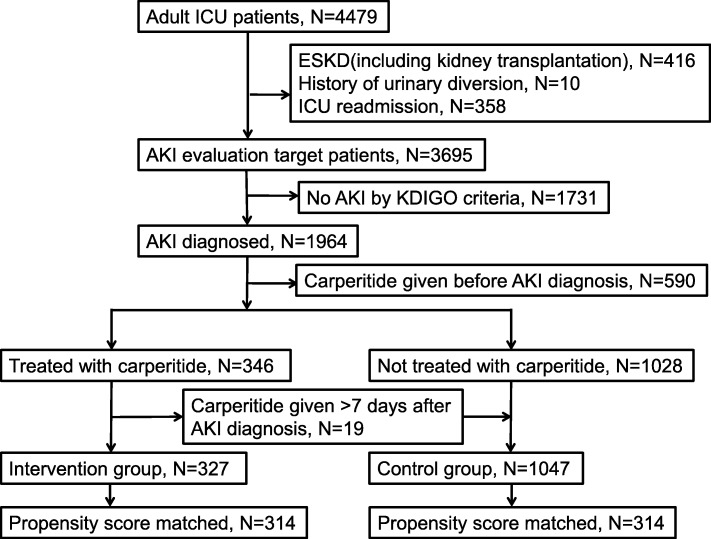
Flow chart for study patients. *AKI* acute kidney injury, *ESKD* end-stage kidney disease, *ICU* intensive care unit, *KDIGO* Kidney Disease: Improving Global Outcomes

During the eight years of the study period, 4479 adult patients were admitted to the ICU. After excluding patients with ESKD including kidney transplantation, those with a history of urinary diversion, those with ICU readmission, those with no AKI by KDIGO criteria, and those treated with carperitide before AKI diagnosis, we included 1374 patients in this study (Fig. 1). Among these patients, 346 were treated with carperitide during their ICU stay. Because 19 patients were treated with carperitide > 7 days after AKI diagnosis, the final treatment group consisted of 327 patients (23.8%), and the control group consisted of 1047 patients (76.2%). Patient demographic and clinical data during ICU stay are listed in Table 1. The treatment group was older (71 versus 67 years; *p* < 0.001) and had more hematologic disease (7.3% versus 2.6%; *p* < 0.001). All serum creatinine levels (at baseline, ICU admission, and AKI diagnosis) were greater in the treatment group than in the control group (76 versus 73, 107 versus 81, 123 versus 83 μmol/L respectively; *p* < 0.001 for all comparisons).

**Table 1:** Patient demographic characteristics

Characteristic	Overall	Control group	Treatment group	*p* value
Number of patients	1374	1047	327	
Age (years)	68 (57–76)	67 (55–76)	71 (61–78)	< 0.001
Male sex	919 (66.9)	702 (67.0)	217 (66.4)	0.818
Height (cm)	164 (156–169)	164 (156–169)	164 (156–169)	0.59
Body weight (kg)	59 (50–68)	59 (50–68)	58 (50–68)	0.762
ICU admission route				0.055
OR (elective)	404 (29.4)	291 (27.8)	113 (34.6)	
OR (emergency)	223 (16.2)	177 (16.9)	46 (14.1)	
Emergency department	336 (24.5)	270 (25.8)	66 (20.2)	
Ward	375 (27.3)	284 (27.1)	91 (27.8)	
Other hospital	36 (2.6)	25 (2.4)	11 (3.4)	
Comorbidity				
Hematologic disease	51 (3.7)	27 (2.6)	24 (7.3)	< 0.001
Metastatic cancer	49 (3.6)	39 (3.7)	10 (3.1)	0.57
Immunosuppression	123 (9.0)	86 (8.2)	37 (11.3)	0.086
Liver failure	39 (2.8)	26 (2.5)	13 (4.0)	0.156
Primary damaged organ				0.117
Cardiovascular	550 (40.0)	423 (40.4)	127 (38.8)	
Respiratory	237 (17.2)	181 (17.3)	56 (17.1)	
Digestive	239 (17.4)	187 (17.9)	52 (15.9)	
Neurologic	212 (15.4)	147 (14.0)	65 (19.9)	
Other	136 (9.9)	109 (10.4)	27 (8.3)	
Infection at ICU admission	254 (18.5)	201 (19.2)	53 (16.2)	0.224
NPPV	147 (10.7)	108 (10.3)	39 (11.9)	0.411
MV duration (h) (*n* = 837)	17.5 (10.0–68.8)	17.5 (10.3–64.9)	16.6 (8.9–87.7)	0.821
APACHE II score	18 (14–22)	18 (14–22)	18 (14–23)	0.384
Serum creatinine (μmol/L)				
Baseline	73 (63–86)	73 (62–82)	76 (66–99)	< 0.001
ICU admission	87 (65–134)	81 (61–122)	107 (81–152)	< 0.001
AKI diagnosis	90 (65–141)	83 (60–126)	123 (88–171)	< 0.001
ICU-AKI (h)	12.7 (7.4–25.0)	13.9 (7.7–27.6)	10.1 (6.7–15.6)	< 0.001

Data are presented as no. (%) or as median (interquartile range; 25th–75th percentile)

*AKI* acute kidney injury, *APACHE II* Acute Physiology and Chronic Health Evaluation II, *ICU* intensive care unit, *ICU-AKI* h duration between ICU admission and AKI diagnosis, *MV* mechanical ventilation, *NPPV* noninvasive positive-pressure ventilation, *OR* operating room

**Fig. 2:**
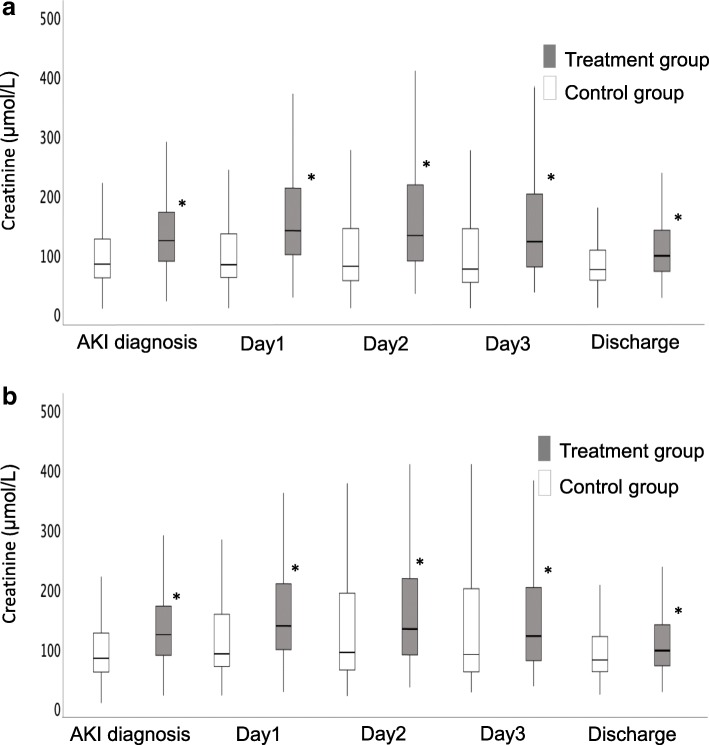
Box plot for trends in serum creatinine levels at Day 1, Day 2, and Day 3 after AKI diagnosis and at ICU discharge for the full cohort (2a) and propensity-matched cohort (2b). *AKI* acute kidney injury. Gray box: treatment group, White box: control group

**Fig. 3:**
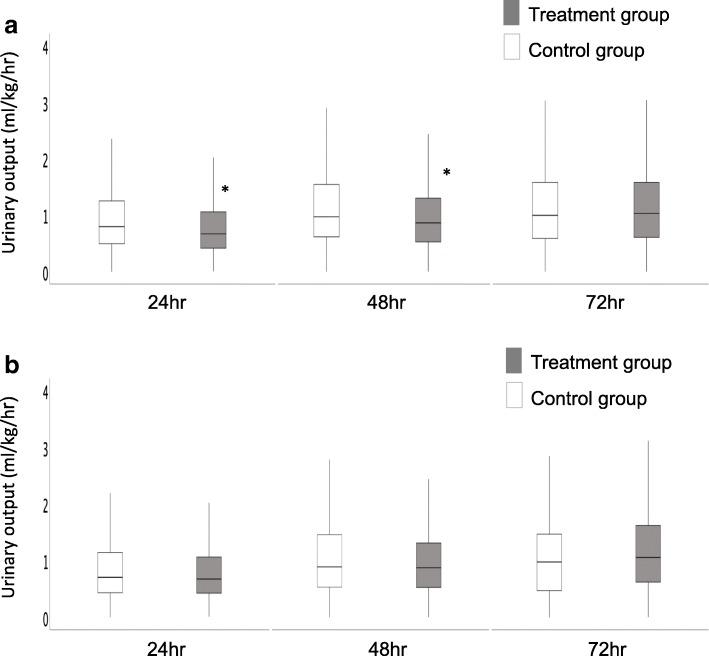
Box plot for trends in urinary output at 24 h, 48 h, and 72 h after AKI diagnosis for the full cohort (3a) and propensity-matched cohort (3b). Gray box: treatment group, White box: control group

The creatinine level trend for the first 3 days and at discharge and the urinary output trend for the first 72 h after AKI diagnosis for the full cohort are shown in Figs. 2a and 3a, respectively. Creatinine levels were significantly greater for all days in the treatment group compared with the control group (*p* < 0.001). Urinary output was significantly greater at 24 h (*p* < 0.001) and 48 h (*p* = 0.003) in the control group compared with the treatment group. It was not different between the two groups at 72 h (*p* = 0.392).

**Fig. 4:**
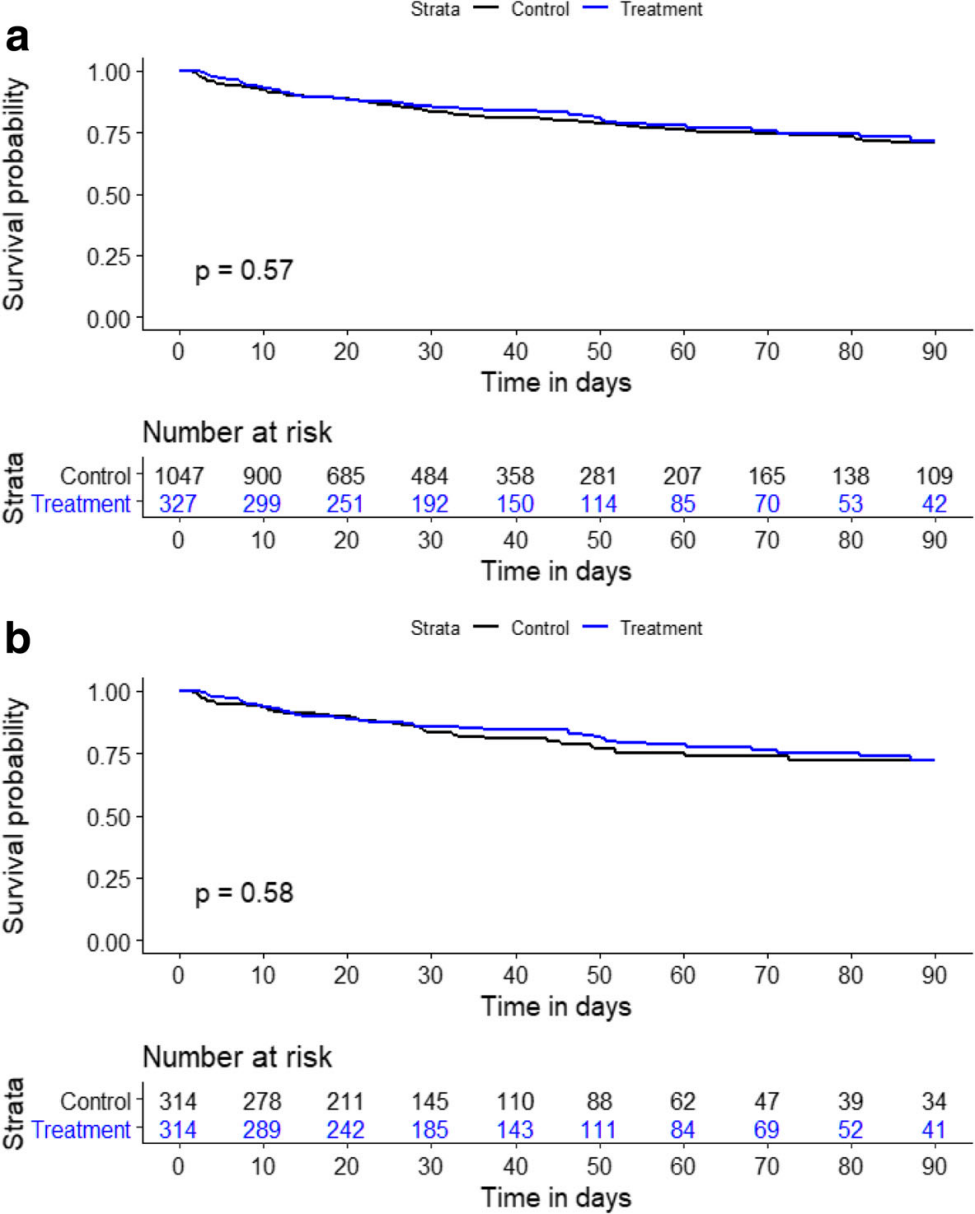
Kaplan-Meier survival curves by the treatment group for the full cohort (4a) and propensity-matched cohort (4b) over 90 days

Table 2 shows patient outcomes for the full cohort. The primary outcome was observed more frequently in the treatment group than in the control group (29.7% versus 23.4%; *p* = 0.022). The ICU LOS (5.5 versus 2.8 days; *p* < 0.001) and hospital LOS (44 versus 34 days; *p* < 0.001) were also significantly longer in the treatment group. Values for ICU mortality and hospital mortality did not differ between the 2 groups (10.7% versus 9.1%; *p* = 0.379 and 22.6% versus 19.7%; *p* = 0.247, respectively). Use of RRT during the ICU stay was required significantly more often (17.1% versus 10.1%; *p* = 0.001), and the highest AKI stages (AKI max-urine output, AKI max-creatinine, AKI max) were significantly worse in the treatment group. Figure 4a shows the Kaplan-Meier survival curves for the full cohort at 90 days. There was no difference in the survival curves between the two groups (*p* = 0.574). The median dose of carperitide was 0.019 μg kg^− 1^ min^− 1^ (interquartile range [IQR], 0.012–0.036 μg kg^− 1^ min^− 1^), and the carperitide infusion period was 2.05 days (IQR, 0.85–3.63 days). The median time from AKI diagnosis to carperitide administration was 0.65 days (IQR, 0.19–1.32 days).

**Table 2:** Patient outcomes

Variable	Overall (*n* = 1374)	Control group (*n* = 1047)	Treatment group (*n* = 327)	*p* value
Primary outcome	342 (24.9)	245 (23.4)	97 (29.7)	0.022
In-hospital mortality	280 (20.4)	206 (19.7)	74 (22.6)	0.247
RRT dependence	33 (2.4)	24 (2.3)	9 (2.8)	0.635
No AKI recovery	183 (13.3)	129 (12.3)	54 (16.5)	0.051
AKI max-urine output				< 0.001
No AKI	254 (18.5)	199 (19.0)	55 (16.8)	
Stage 1	544 (39.6)	448 (42.8)	96 (29.4)	
Stage 2	403 (29.3)	279 (26.6)	124 (37.9)	
Stage 3	173 (12.6)	121 (11.6)	52 (15.9)	
AKI max-creatinine				< 0.001
No AKI	577 (42.0)	523 (50.0)	54 (16.5)	
Stage 1	405 (29.5)	280 (26.7)	125 (38.2)	
Stage 2	145 (10.6)	87 (8.3)	58 (17.7)	
Stage 3	247 (18.0)	157 (15.0)	90 (27.5)	
AKI max				< 0.001
Stage 1	635 (46.2)	538 (51.4)	97 (29.7)	
Stage 2	436 (31.7)	312 (29.8)	124 (37.9)	
Stage 3	303 (22.1)	197 (18.8)	106 (32.4)	
RRT during ICU stay	162 (11.8)	106 (10.1)	56 (17.1)	0.001
ICU LOS (days)	3.4 (1.9–6.6)	2.8 (1.8–5.6)	5.5 (3.6–9.6)	< 0.001
ICU mortality	130 (9.5)	95 (9.1)	35 (10.7)	0.379
Hospital LOS (days)	37 (21–66)	34 (20–62)	44 (26–76)	< 0.001
Dialysis-free survival	1070 (77.9)	823 (78.6)	247 (75.5)	0.243

Data are presented as no. (%) or as median (interquartile range; 25th–75th percentile)

*AKI* acute kidney injury, *AKI-max* worst stage of AKI, *ICU* intensive care unit, *LOS* length of stay, *No AKI* recovery creatinine level ≥ 200% of baseline at hospital discharge, *RRT* renal replacement therapy

After PS matching, 314 patients from each group were matched, and the patient characteristics were well-balanced (Table 3). The creatinine level trend for the first 3 days and at hospital discharge and the urinary output trend for the first 72 h after AKI diagnosis for the PS-matched population are shown in Figs. 2 and 3b and b, respectively. Creatinine levels were significantly greater for the 3 days and ICU discharge in the treatment group compared with the control group (*p* < 0.001 for Day 1, Day 2, and ICU discharge; *p* = 0.010 for Day 3). However, urinary output did not differ between the 2 groups for all observation periods (*p* = 0.753, *p* = 0.468, *p* = 0.064, respectively).

**Table 3:** Demographic characteristics for propensity-matched patients

Characteristic	Control group	Treatment group	SMD
Number of patients	314	314	
Age (years)	68 (14)	68 (14)	0.006
Male sex	33 (47)	33 (47)	< 0.001
Height (cm)	163 (9)	163 (9)	0.012
Body weight (kg)	59 (14)	60 (14)	0.048
ICU admission route			
OR (elective)	118 (37.6)	109 (34.7)	0.06
OR (emergency)	40 (12.7)	46 (14.6)	0.056
Emergency department	70 (22.3)	64 (20.4)	0.047
Ward	77 (24.5)	84 (26.8)	0.051
Other hospital	9 (2.9)	11 (3.5)	0.036
Comorbidity			
Hematologic disease	19 (6.1)	16 (5.1)	0.042
Metastatic cancer	9 (2.9)	10 (3.2)	0.019
Immunosuppression	36 (11.5)	31 (9.9)	0.052
Liver failure	12 (3.8)	12 (3.8)	< 0.001
Primary damaged organ			
Cardiovascular	129 (41.1)	125 (39.8)	0.026
Respiratory	57 (18.2)	53 (16.9)	0.034
Digestive	46 (14.6)	51 (16.2)	0.044
Neurologic	57 (18.2)	60 (19.1)	0.025
Other	25 (8.0)	25 (8.0)	< 0.001
Infection at ICU admission	53 (16.9)	50 (15.9)	0.026
NPPV	35 (11.1)	37 (11.8)	0.020
MV duration (h) (*n* = 165)	73.9 (15.0)	53.0 (9.5)	0.069
APACHE II score	19 (7)	19 (7)	0.006
Serum creatinine (μmol/L)			
Baseline	92 (74.3)	91 (46.9)	0.012
ICU admission	150 (172.4)	137 (99.9)	0.092
AKI diagnosis	149 (158.2)	148 (99.9)	0.007
AKI stage at diagnosis			
Stage 1	258 (82.2)	250 (79.6)	0.064
Stage 2	29 (9.2)	37 (11.8)	0.083
Stage 3	27 (8.6)	27 (8.6)	< 0.001
ICU-AKI (h)	14.9 (0.7)	13.9 (0.7)	0.063

Data are presented as no. (%) or as mean (standard deviation)

*AKI* acute kidney injury, *APACHE II* Acute Physiology and Chronic Health Evaluation II, *ICU* intensive care unit, *ICU-AKI* h duration between ICU admission and AKI diagnosis, *OR* operating room, *SD* standard deviation, *SMD* standard mean difference

Significant difference of the primary outcome, as seen with the full cohort, was not detected in the matched cohort, although the value for the treatment group was numerically greater (29.0% versus 25.2%; *p* = 0.281) (Table 4). ICU mortality and hospital mortality were also similar between the 2 groups (10.5% versus 8.0%; *p* = 0.270, 22.0% versus 19.1%; *p* = 0.374, respectively). However, ICU LOS (5.6 versus 2.7 days; *p* < 0.001) and hospital LOS (44 versus 35 days; *p* = 0.001) were significantly longer in the treatment group. Use of RRT during the ICU stay was similarly required in the 2 groups (16.6% versus 14.0%; *p* = 0.375). The AKI max-urine output was also similar between the 2 groups. However, AKI max-creatinine and AKI max were significantly worse in the treatment group. Figure 4b shows the Kaplan-Meier survival curves for the propensity-matched cohort at 90 days. There was no difference in the survival curves between the two groups (*p* = 0.575). The median dose of carperitide was 0.019 μg kg^− 1^ min^− 1^ (interquartile range [IQR], 0.012–0.036 μg kg^− 1^ min^− 1^), and the carperitide infusion period was 2.11 days (IQR, 0.90–3.61 days). The median time from AKI diagnosis to carperitide administration was 0.68 days (IQR, 0.19–1.33 days).

**Table 4:** Outcomes for propensity-matched patients

Variable	Overall (*n* = 628)	Control group (*n* = 314)	Treatment group (*n* = 314)	*p* value
Primary outcome	170 (27.1)	79 (25.2)	91 (29.0)	0.281
In-hospital mortality	129 (20.5)	60 (19.1)	69 (22.0)	0.374
RRT dependence	22 (3.5)	13 (4.1)	9 (2.9)	0.385
No AKI recovery	93 (14.8)	42 (13.4)	51 (16.2)	0.312
AKI max-urine output				0.123
No AKI	120 (19.1)	66 (21.0)	54 (17.2)	
Stage 1	202 (32.2)	110 (35.0)	92 (29.3)	
Stage 2	212 (33.8)	95 (30.3)	117 (37.3)	
Stage 3	94 (15.0)	43 (13.7)	51 (16.2)	
AKI max-creatinine				< 0.001
No AKI	186 (29.6)	135 (43.0)	51 (16.2)	
Stage 1	213 (33.9)	92 (29.3)	121 (38.5)	
Stage 2	89 (14.2)	31 (9.9)	58 (18.5)	
Stage 3	140 (22.3)	56 (17.8)	84 (26.8)	
AKI max				< 0.001
Stage 1	236 (37.6)	142 (45.2)	94 (29.9)	
Stage 2	225 (35.8)	105 (33.4)	120 (38.2)	
Stage 3	167 (26.6)	67 (21.3)	100 (31.8)	
RRT during ICU stay	96 (15.3)	44 (14.0)	52 (16.6)	0.375
ICU LOS (days)	3.8 (2.3–6.9)	2.7 (1.7–4.8)	5.6 (3.7–9.6)	< 0.001
ICU mortality	58 (9.2)	25 (8.0)	33 (10.5)	0.27
Hospital LOS (days)	40 (23–69)	35 (21–62)	44 (26–76)	0.001
Dialysis-free survival	482 (76.8)	243 (77.4)	239 (76.1)	0.706

Data are presented as no. (%) or as median (interquartile range; 25th–75th percentile)

*AKI* acute kidney injury, *AKI max* worst stage of AKI, *ICU* intensive care unit, *LOS* length of stay, *MV* mechanical ventilation, *No AKI* recovery creatinine level ≥ 200% of baseline at hospital discharge, *NPPV* noninvasive positive-pressure ventilation, *RRT* renal replacement therapy

As for the primary outcome, additional analysis using IPTW to fit a marginal structural model confirmed the robustness of the finding (risk difference, 0.8%; 95%CI, − 5.6 to 7.1).

## Discussion

### Key findings

In this study using PS matching, we studied the effect of low-dose ANP administration on a clinically important outcome, the composite of hospital mortality, RRT dependence at hospital discharge, and no AKI recovery, in general critically ill patients who developed AKI in the ICU. We found that the primary outcome was not affected by carperitide administration. Moreover, the highest AKI stage was worse in patients who were treated with carperitide, and ICU LOS and hospital LOS were longer in the carperitide treatment group.

### Comparison to previous studies

In the medical literature, there are five studies [31, 40–43] that have assessed the therapeutic effects of ANP; three studies used carperitide [31, 42, 43] and two studies used anaritide [40, 41]. Of the three meta-analyses, one meta-analysis pooled the effect of carperitide, anaritide, and urodilatin all together [14] and the other two focused on carperitide [15, 16]. As carperitide is the only ANP that is commercially available in Japan, we assessed the effect of carperitide in this study.

To evaluate the therapeutic effect of anaritide (a 25-amino-acid synthetic form of ANP), two large-scale randomized controlled trials (RCTs) were conducted in 1990s [40, 41]. Allgren et al. conducted a multicenter RCT of anaritide in 504 critically ill patients with acute tubular necrosis [40]. Study patients received a 24-h infusion of either anaritide (0.2 μg kg^− 1^ min^− 1^) or placebo. Although anaritide did not improve the overall rate of dialysis-free survival at 21 days after treatment, dialysis-free survival was improved in the anaritide group compared with the placebo group in the prospectively defined subgroup of 120 patients with oliguria (< 400 mL/day; 27% versus 8%; *p* = 0.008). On the basis of this subgroup analysis, they conducted a confirmatory double-blind, multicenter RCT in patients with oliguric AKI [41]. However, they did not find a significant difference in dialysis-free survival (21% versus 15%; *p* = 0.22). It has been suggested that the high dose of ANP administered in those studies induced hypotension, which might have offset the therapeutic effect of ANP [14]. In our study, carperitide was administered in low doses; however, it did not improve renal function or RRT requirement or prognosis either.

Since 2000, two RCTs have examined the therapeutic effect of low-dose ANP on AKI [42, 43]. One study (0.05 μg kg^− 1^ min^− 1^; *N* = 61) showed that 21% of patients who underwent cardiac surgery in the ANP group required dialysis before or at day 21 compared with 47% in the placebo group (*p* = 0.009) [42]. The other RCT (0.02 μg kg^− 1^ min^− 1^; *N* = 77) also studied patients who underwent cardiovascular surgery and showed that, although ANP increased urine output, it did not significantly improve renal function or RRT requirement compared with placebo [43]. Both RCTs were of low quality with small sample size, and studied only patients undergoing cardiovascular surgery. In addition, one study was conducted before consensus definitions of AKI were developed [42].

Moreover, although three of the four RCTs have dialysis-free survival at 21 days after treatment on the primary outcome [40–42], one RCT has made the renal outcome changes such as creatinine change and urine volume change on the primary outcome [43]. We adopted patient-centered outcome as the primary outcome which was the composite of hospital mortality, the need of RRT or no recovery from AKI at hospital discharge.

With respect to general ICU patients who developed AKI, to the best of our knowledge, there is only one observational study that evaluated the therapeutic effect of low-dose ANP (0.028 μg kg^− 1^ min^− 1^), which found no therapeutic effect of ANP [31]. Although that study was a multicenter, prospective, observational study, the number of patients treated with ANP was small (*N* = 63), suggesting a lack of power to detect significance. Although the present study was a single-center study, the number of patients treated with low-dose ANP (0.019 μg kg^− 1^ min^− 1^) was more than 300, the largest sample size among all low-dose ANP studies [31, 42, 43].

### Significance and implications

Previous five studies examining the therapeutic effects of any dose of ANP found inconsistent benefits [17–30]. The KDIGO clinical practice guideline for AKI suggests not using ANP to treat AKI and requires further trials of ANP at low doses [33]. Recent meta-analysis focusing on low-dose ANP implied its beneficial therapeutic effects in patients with cardiac surgery [16]; however, two recent observational studies, including the present study, found that low-dose ANP did not change the outcome of critically ill patients who developed AKI [31]. The difference might be due to patient background (cardiovascular versus general ICU patients), or study design (randomized versus observational). Although ANP administration is pharmacologically effective in increasing the GFR [9], this effect might be offset by a hypotensive side effect [31]. Critically ill patients with AKI (e.g., septic shock) might be more prone to the vasodilative effect of low-dose ANP compared to patients with ANP administered prophylactically or those with less critical conditions (e.g., elective cardiovascular surgery) [16, 42]. Although the two recent studies in critically ill patients were observational studies, the lack of effectiveness of ANP causes a stir on the use of ANP for AKI in the critically ill.

### Study strengths and limitations

The present study has several strengths. The number of patients who were treated with ANP was the largest among all low-dose ANP studies [31, 42, 43]. The findings will be more applicable to patients in the ICU than those from previous studies in cardiac surgery patients. Furthermore, we evaluated physiological outcomes (changes in serum creatinine levels and urine output) as well as patient-centered outcomes (composite of mortality, RRT dependence, and nonrecovery of renal function at hospital discharge). Because of the limited evidence in the literature for a therapeutic effect of low-dose ANP, the present study should provide valuable information with respect to understanding the use of ANP for the management of AKI in the ICU.

The present study also has several limitations. First, this was a single-center study whose results might be influenced by the local clinical practice and may limit the generalizability of its findings. Second, we did not collect data on blood pressure or use of vasopressors. Third, although we used PS matching to minimize the bias due to confounding factors, the results of PS matching are generalizable only among those in the range of PS values included in the analysis, and they may not be applicable to those who are out of this range. Moreover, there might have been unmeasured confounders that were not addressed in the PS model.

## Conclusions

In the present retrospective observational study in critically ill patients with AKI, we found no therapeutic effect of low-dose ANP. Considering the currently available evidence, ANP should not be used to treat AKI in critically ill patients.

## Data Availability

The study data supporting the conclusions of this article are available from the corresponding author. The study data will be provided by request.

## Abbreviations

AKI: Acute kidney injury
ANP: Atrial natriuretic peptide
APACHE: Acute Physiology and Chronic Health Evaluation
CIAKI: Contrast-induced AKI
CKD: Chronic kidney disease
GFR: Glomerular filtration rate
ICU: Intensive care unit
KDIGO: Kidney Disease: Improving Global Outcomes
MAP: Mean arterial pressure
PS: Propensity score
RCT: Randomized controlled trial
RRT: Renal replacement therapy
SAPS: Simplified Acute Physiology Score
